# FISH and chips: a review of microfluidic platforms for FISH analysis

**DOI:** 10.1007/s00430-019-00654-1

**Published:** 2020-01-21

**Authors:** Pablo Rodriguez-Mateos, Nuno Filipe Azevedo, Carina Almeida, Nicole Pamme

**Affiliations:** 1 Department of Chemistry and Biochemistry, University of Hull, Cottingham Road, Hull, HU6 7RX UK; 2 LEPABE-Laboratory for Process Engineering, Environment, Biotechnology and Energy, Department of Chemical Engineering, Faculty of Engineering of University of Porto, Rua Dr. Roberto Frias, s/n, 4200-465 Porto, Portugal; 3 Biomode SA, Av. Mestre José Veiga, 4715-330 Braga, Portugal; 4 INIAV, I.P.-National Institute for Agricultural and Veterinary Research, Rua dos Lagidos, Lugar da Madalena, Vairão, 4485-655 Vila Do Conde, Portugal; 5 CEB-Centre of Biological Engineering, University of Minho, 4710-057 Braga, Portugal

**Keywords:** Microfluidics-assisted FISH, µFISH, Microfluidics, Lab-on-a-Chip (LOC), Fluorescence in situ hybridization (FISH)

## Abstract

Fluorescence in situ hybridization (FISH) allows visualization of specific nucleic acid sequences within an intact cell or a tissue section. It is based on molecular recognition between a fluorescently labeled probe that penetrates the cell membrane of a fixed but intact sample and hybridizes to a nucleic acid sequence of interest within the cell, rendering a measurable signal. FISH has been applied to, for example, gene mapping, diagnosis of chromosomal aberrations and identification of pathogens in complex samples as well as detailed studies of cellular structure and function. However, FISH protocols are complex, they comprise of many fixation, incubation and washing steps involving a range of solvents and temperatures and are, thus, generally time consuming and labor intensive. The complexity of the process, the relatively high-priced fluorescent probes and the fairly high-end microscopy needed for readout render the whole process costly and have limited wider uptake of this powerful technique. In recent years, there have been attempts to transfer FISH assay protocols onto microfluidic lab-on-a-chip platforms, which reduces the required amount of sample and reagents, shortens incubation times and, thus, time to complete the protocol, and finally has the potential for automating the process. Here, we review the wide variety of approaches for lab-on-chip-based FISH that have been demonstrated at proof-of-concept stage, ranging from FISH analysis of immobilized cell layers, and cells trapped in arrays, to FISH on tissue slices. Some researchers have aimed to develop simple devices that interface with existing equipment and workflows, whilst others have aimed to integrate the entire FISH protocol into a fully autonomous FISH on-chip system. Whilst the technical possibilities for FISH on-chip are clearly demonstrated, only a small number of approaches have so far been converted into off-the-shelf products for wider use beyond the research laboratory.

## Introduction

In situ hybridization (ISH) is a molecular technique in which a nucleic acid sequence of interest within an intact cell or a tissue section is hybridized with a labeled probe to give a measurable signal. In situ hybridization was first demonstrated in 1969 by Gall and Pardue in the cytogenetic field using radioactive rRNA probes for localizing and quantifying nucleic acid targets in the toad *Xenopus* [1]. In 1975, Manning et al. carried out the first non-radioisotopic ISH using rRNA probes attached to 60-nm particles via biotin–avidin binding for mapping genes in *Drosophila melanogaster* [2]. The prospect of ISH-based techniques changed in 1980, when Bauman et al. took advantage of covalent binding of commercially available fluorochromes to RNA, allowing fluorescence microscopy to be used for visualization, coining the term fluorescence in situ hybridization (FISH) [3]. With improvements in fluorescence microscopy and fluorescent labels for a variety of nucleic acid probes, FISH assays have been developed extensively during the last decades and have made a considerable impact on biotechnology, genomics and bioinformatics [4, 5]. Nowadays, a range of nucleic acid probes, and even probes made of nucleic acid mimics, are commercially available to localize and quantify specific sequences of RNAs, genes and entire chromosomes [6–9].

FISH is powerful since it allows not only pinpointing the precise location of molecules of interest within a cell population or tissue slice with single cell resolution, but also quantification on a cell-by-cell basis [10]. FISH has been applied to detect and localize the presence or absence of specific genes within chromosomes for diagnosis of chromosomal abnormalities [4], as well as to cancer prognosis [4, 11–13], and to quantitatively study the spatial–temporal patterns of gene expression within cells and tissues [14]. FISH is also used for species identification [15–17] and to study microbial diversity in complex samples [5, 17]. A particularly well-known use of FISH has been in status assessment of the human epidermal growth factor 2 (HER2) gene as a prognostic biomarker, overexpressed in some individuals with breast and gastric cancer [12, 13]. HER2-targeted therapies can improve the survival rate of patients [18], and FISH is a standard and recommended technique to routinely detect HER2 overexpression by counting the number of HER2 gene *loci* in a cell nucleus and comparing it to the number of centromeres in the chromosome 17 (Cen17), where it is located [19–21]. The successful development of FISH for mammalian cells paved the way to applications in microbial cells [15–17]. Targeting microorganisms, however, poses a set of challenges, due to their diverse cellular structures and cell wall properties. Thus, quite often, FISH protocols have to be modified for each target microorganism. Furthermore, a wider range of probe molecules have been introduced including synthetic molecules that mimic natural nucleic acids, such as peptide nucleic acids (PNA). These have improved the performance of FISH in terms of time-to-result and signal intensity [22, 23].

FISH can be applied to a range of samples: mammalian cells or patient tissue samples are studied frequently, microbial populations in food or environment samples are also of interest [10, 24, 25]. Depending on the type of sample, the targeted sequences and the type of probe used, FISH assays protocols will be different. However, all FISH assays generally follow a number of common steps: (1) *Cell or tissue preparation*. Cells are either immobilized on a glass slide or, less frequently, left in suspension. Tissues are fixed and sliced and placed on a microscopy glass slide. The complexity and duration of these steps depend a lot on the sample. For instance, for microbial cells, this can take a few minutes and simply involve flaming the sample to immobilize cells on a glass slide; or it can take a few days for tissue biopsy samples that undergo a long paraffinization, sectioning and deparaffinization process that aims to provide thin and stable sections of tissue for the FISH analysis. (2) *Enzymatic digestion*. In case of targeting chromosomal DNA in mammalian cells, a proteinase digestion is performed to remove cytoplasmatic and chromosomal proteins to improve the access to the DNA in the cell nucleus. For bacteria, the use of enzymatic treatments is also common; in this case, they are used after the fixation step to improve cell wall permeability and thus facilitating probe penetration. Then, (3) *fixation* and *dehydration* of the cells is carried out in a series of paraformaldehyde and/or ethanol treatments. This stops any metabolic activity and maintains the cellular structure. (4) Next, the cells are *hybridized* with the fluorescent nucleic acid probe, often at 37 °C, sometimes at higher temperatures of around 50–60 °C. This hybridization step is generally the longest in the FISH protocol, taking several hours or sometimes overnight, since sufficient time must be given to allow the probe to penetrate the cell membrane and find its way by diffusion to the correct location within the cell for hybridization. The probe solution is often rather viscous, which further slows down diffusion. The required hybridization time and temperature will depend on the targeted cell and on the type of probe being used. For instance, when targeting chromosomes, an overnight hybridization step will often be needed; whereas for bacteria, especially when using synthetic probes, the hybridization step can be as short as 15 min [4, 5]. (5) Following hybridization, any excess and unbound fluorescent probes must be thoroughly *washed*. Finally, (6) cells are *imaged* via fluorescence microscopy, often with large magnification objectives (60–100x), so that individual cell nuclei can be resolved on the glass slide.

FISH offers advantages compared to other molecular techniques, such as the preservation of cell morphology and cell integrity. There is no requirement for nucleic acid amplification, which often can introduce bias into the final result, either due to the amplification of extracellular DNA (usually from dead cells) or, even, artifacts, if amplification conditions are not properly set. Also, amplification polymerases are prone to inhibition by several molecules present in biological samples [26–28]. However, FISH protocols are generally time consuming, labor intensive and relatively costly [4, 5] due to the large number of fixing, incubation and washing steps, especially the long probe hybridization times, and also the lack of automation, the cost associated with the probes and reagents and the need for well-trained personnel. Pre-enrichment steps required for some of the clinical and food diagnostics applications further lengthen the protocols. These challenges have slowed widespread utilization of FISH in clinical or diagnostic settings.

Transferring the FISH protocol onto microfluidic, lab-on-a-chip platforms may offer an avenue to address these challenges. Microfluidics concerns the shrinking down of liquid handling into sub-millimeter channels, with µL or even nL internal volumes, onto small footprint glass or polymer devices. This increases mass transport and heat dissipation, allows precise spatial and temporal control of the cell microenvironment and lends itself to the integration of all sample processing setups onto one device. The reader is guided to excellent reviews of the general field [29–32]. Microfluidic devices are now widespread in bioanalysis and clinical diagnostics, including protein, nucleic acid, cell and tissue analysis [30].

In recent years, a variety of approaches for lab-on-chip-based FISH assays have been demonstrated at proof-of-concept stage, aiming to reduce assay time, reagent volumes and facilitate automation. The level of integration of the FISH procedure, the type of target cells and strategies to immobilize them differ significantly among the published studies. Some have tried to use simple devices that interface with existing equipment and workflows, and others have tried to move towards an integrative approach aiming to perform the entire FISH protocol in a fully autonomous FISH on-chip system. In the following, we review the different microfluidic platforms and approaches for carrying out FISH assays on-chip. We significantly extend on earlier reviews by Kwasny et al. on microfluidic FISH for chromosome abnormalities [33], by Sato on microfluidic FISH for analysis of circulating tumor cells [34] and a historic FISH review with microfluidic FISH outlook by Huber et al. [35]. Here, we include microfluidic platforms across the full range of samples, from cells to tissue, from mammalian to microbial samples. The review is structured by design approaches for cell- and tissue-based FISH, generally moving from simple channel networks towards more complex systems that aim to integrate the entire FISH protocol in a standalone device. The wide array of approaches reviewed is summarized in Table 1.

**Table 1:** Comparison of FISH-on-chip methods based on design approach, sample and application, volume of hybridization probe needed, time and temperature for hybridization, steps carried out off-chip and overall level of integration and automation

Device	Device features	Sample	Probe solution	Hybridization	Off-chip preparation	Level of automation	Refs.
*Straight channels for cell capture*							
Microchip array	Glass device of microscope slide size with ten straight channels (310 µm wide, 55 µm deep) 50 mm long, wells at either end (1.5 µL), 170 µm thick cover plate to seal	PBMCs (chromosomal abnormalities in MM)	1 µL	4 h (37 °C)	Cell suspension	User pipettes and applies vacuum, some automated electrokinetic transport	[37]
microFIND^®^	PDMS microchannel (300 µm wide, 50 µm deep) atop TiO_2_ coated glass slide	Human Daudi, Jurkat, NB4, Raji and U937 cells, (sex chromosomes and oncohematology)	0.3 µL	overnight (37 °C)	Cell suspension	User assembles device, pipettes and aspirates	[38]
FISHing line	Channels (40 µm wide, 50 µm deep) etched into microscope glass slide, sealed with adhesive tape	K567 and Jurkat cells (MRD analysis)	0.2 µL	2 h (37 °C)	Cell fixing	User pipettes liquids, attaches/removes adhesive	[41]
*Chambers for cell capture*							
Deep chamber	COC device with narrow channels (60 µm wide, 30 µm deep) into and out of deep chamber (1 mm bottom diameter, 380 µm deep)	Breast cancer cell lines (HER2, ERBB2)	2.5 µL	overnight (37 °C)	Cell suspension	Automated pump system	[43]
OncoCEE™	PDMS chamber (12 mm wide × 55 µm deep) with 9000 streptavidin-coated posts of between 75 and 150 µm diameter atop microscope coverslip	CTC from peripheral blood and bone marrow (HER2)	n/a	n/a	RBC removal, CTC enrichment, incubation with biotinylated Ab	Test performed to order by company	[44–46, 81]
Metaphase spreads	Glass coverslip with double sided tape (50 µm), initially interfaced with PMMA open splashing chamber, then with PDMS flow cell	Peripheral blood lymphocytes (X chromosome)	5 µL	overnight (37 °C)	Expansion, hypotonic treatment, fixation	Manual interchange of splashing and flow device, user changes over tubing	[47, 48]
*Micrometer sized filters and capture elements*							
Microarray (10 × 10) in PET	PDMS device with top and bottom channel sandwiched around a PET micromesh featuring a 10 × 10 array of microcavities, each 2 µm in diameter and spaced at 30 µm distance	Raji Burkitt’s lymphoma cells, (β-actin mRNA)	1 µL	2 h (42 °C)	Cell fixation	User operates pump, tubing and connectors	[52]
Microhole-array chip (35 × 35)	Silicon nitride membranes featuring a 35 × 35 microhole array of 5 µm diameter holes. Polycarbonate adapter for the fluidics	Human retina pigment epithelia cells ARPE-19 (EGFR)	2 µL	14–20 h	Cell suspension	Automated software analysis	[53, 54]
Celsee™	Glass device with channel network (75 µm deep) leading to 56 k traps (each 20 × 25 µm sides, 30 µm deep) featuring pore channels (9 µm wide) leading to outlet channel below	CTCs from peripheral blood (HER2)	Five drops	overnight (37 °C)	Partial fixation	Entirely automated, user adds reagents to platform	[55, 56]
Track etched membrane	5 µm pore diameter membrane sandwiched between channels formed in double sided tape (2 mm wide, 4 mm long, 100 µm deep). Glass slip at bottom, acrylic sheet with access holes at top	MDCK cells infected with influenza (viral RNA)	40 µL	5 min (37 °C)	Cell fixation	Automated pump protocol and image processing, user adds reagents to platform	[57]
In-line weirs	Silicon device with weirs of 10 µm width and 1–2 µm gaps between them, covered with glass plate	*G. lamblia* cells (18S rRNA)	1 µL	10 min (48 °C)	Cell fixation and permeabilization	Hybridization and washing on device, largely manual operation	[58]
In-line pillars	PDMS microchannel (100 µm wide, 30 µm deep) with three rows of pillars (15 µm wide, 5 µm gaps)	*S. cerevisiae* cells (rRNA)	100 µL	60 min (59 °C)	Cell fixation	User pipettes liquids and operates pump	[59]
Droplet microfluidic chemistrode	PDMS channels (100 µm wide) to generate 10 nL plugs surrounded by immiscible oil, stored in Teflon tubing of 200 µm diameter	*P. curdlanolyticus and E. coli cells* (16S rRNA)	100 µL	2.5 h (48 °C)	Hybridization and washing	Droplet generation and culture of bacterial cells, followed by fixation with EtOH droplets	[61]
Dielectro-phoresis trap	Microchannel (60 mm long, 2.6 mm wide, 10–15 µm deep) with interdigitated electrodes	*E. coli* cells (non-specific bacterial probe)	100 µL	< 30 min (25 °C)	Cell preparation	Manual loading of pumps and tubing	[62]
*Integrated systems towards sample-in-answer-out*							
Circulating microchip	Glass bottom layer with a circular (10 mm diameter) and two straight (580 µm wide) channels at opposite sites (all 40 µm deep), leading to 1.5 µL wells. Flexible middle layer of 0.25 mm PDMS. Rigid glass top layer with control channels	PBMCs (chromosomal abnormalities in MM)	1 µL	4 h (37 °C)	Cell suspension	Automated temperature and actuation control, user loads samples	[37]
Integrated microchip (Backhouse group)	Glass bottom layer with channels (150 µm wide, 50 µm deep) and FISH chamber (2.5 mm diameter), thin Pt film atop for heating, middle layer of flexible PDMS, top layer with air control channels to operate integrated valves	PBMCs (enumeration of X- and Y-chromosomes)	0.5–1 µL	60 min (37 °C)	Cell suspension	Automated temperature, actuation control, user loads samples	[63]
Integrated platform (Lee group)	Glass bottom layer, middle deformable PDMS layer with fluidic channels and reaction chamber (4 mm diameter, 200 µm height), top thick PDMS layer with actuation channels. Device placed on top of two heating blocks	PBMCs and MV4-11 cells (MLL translocation)	0.5 µL	40 min (37 °C)	Cell suspension	Automated temperature, actuation control, user loads samples	[65]
µFlowFISH	Glass device with FISH chamber (120 µm wide) and channel network (60 µm wide), all 20 µm deep, PA gel plugs either site of FISH chamber	*D. vulgaris, Pseudomonas sp.* and *E. coli* cells (16S rRNA)	80 µL	30 min (46–48 °C)	Cell fixation	Automated temperature and actuation control, user performs gel polymerization and loads reagents	[66]
*Tissue analysis*							
Integrated platform (Lee group)	Glass bottom layer, middle deformable PDMS layer with fluidic channels and reaction chamber (5 mm diameter), top thick PDMS layer with actuation channels, device placed on top of two heating blocks	Cancer tissue biopsy slice, 5 mm × 5 mm × 2.5 µm (HER2)	2 µL	16 h (37 °C)	Parafilm embedded tissue	Automated temperature, actuation control, user assembles device and needs to dismantle before microscopy	[67]
*HistoFlex*	Silica substrate, PDMS flow chamber (10 mm × 10 mm × 100 µm), microscope slide top layer, housed in aluminum frame, atop temperature control system	Mouse brain tissue sections 4 µm thick (18S rRNA, miRNA)	20 µL min^−1^ recirculated	15 min (45–50 °C)	Fixation and paraffin embedding	Automated temperature and pumping, manual valve control, user assembles device	[68]
MA-FISH	Microscope slide with tissue slice, 16 mm × 16 mm × 20 µm square chamber from PDMS ‘o-ring’ and spacers, glass layer with branched fluid network to all sides	Breast cancer biopsies 4 µm thick (HER2)	1 µL	4 h (37 °C)	Tissue preparation	Run manually, but pumping heating and image analysis could be automated	[69]
Vertical microfluidic probe	Silicon and glass microfluidic head with microchannels (100 µm × 100 µm and 300 µm × 100 µm) coming to tapered tip	Breast cancer cell line MCF-7 (CEP7 and CEP17)	0.6 µL	3 min (37 °C)	Cell preparation and fixation	Automated probe movement	[75]
		Breast cancer tissue slices (HER2)	105 nL	1–15 min (37 °C)	Tissue sections fully prepared off-chip	Glass slide on heated microscope stage, automated probe movement	[76]

*CTC* circulating tumor cell, *MDCK* Madin–Darby canine kidney, *MM* multiple myeloma, *MRD* minimal residual disease, *PBMC* peripheral blood mononuclear cells, *PDMS* poly dimethyl siloxane, *PA* polyacrylamide, *PC* polycarbonate, *COC* cyclo olefin copolymer

## FISH for cell analysis

To conduct fluorescence in situ imaging on cells within a microfluidic device, a range of issues need to be considered. The cells should be deposited in a single layer on a transparent support, ideally well spread out but not too sparse, to enable easy visual inspection and fast imaging. The device material through which the cells are to be imaged must be optically transparent and must not auto-fluoresce. Furthermore, it must withstand the elevated temperatures and solvent treatments that may be required for the FISH protocol. The thickness of this material has to be compatible with the working distance of the microscope objectives. The effectiveness of reaction, washing and hybridization steps of cells in a chip format depends on the effective transport of reagents to the cells. In the absence of active stirrers or agitators, molecular transport relies on diffusion. For example, for a nucleic acid probe of 25 bp with a diffusion coefficient of 1.58 × 10^−11^ m^2^ s^−1^ [36] to diffuse over a distance of 100 µm, about 5 min are required; for a 1-mm diffusion distance, nearly 9 h are required. For longer probes and viscous probe solutions, these diffusion times will be significantly longer. The diffusion time, thus, needs to be given careful consideration when designing a microfluidic FISH protocol; too large a channel means very long diffusion times and incubation times with little gain from conventional FISH protocols; too small a channel may lead to high back pressures, and thus artificially high shear stresses on the cells, as well as increased likelihood of clogging. To conduct the various steps in the FISH protocol, more than half a dozen different chemical solutions are usually required; these need to be changed over and consideration must be given as to whether an operator performs this manually by changing over vials and tubing or whether to integrate all the fluid handling into an automated system, requiring more complex chip manufacture. The same is true for heating; the designer has to opt between an external heater such as a hotplate already available in the laboratory versus an on-chip integrated heater.

Some researchers have set out to deviate as little as possible from standard laboratory equipment and processes and have, thus, developed relatively simple on-chip FISH systems, with a standard microscope slide at the bottom onto which cells of interest are immobilized and with a simple fluidic channel system atop. Others have opted to developing fully integrated and standalone systems.

### Cells immobilized in single, straight channels

**Fig. 1:**
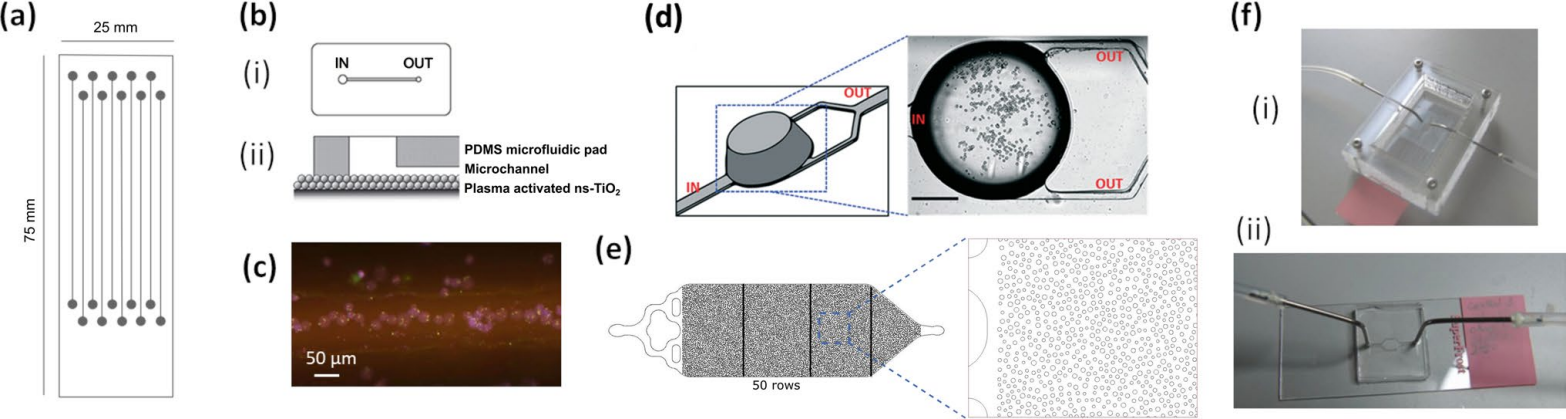
Microfluidic devices with single straight channel systems for cell immobilization for FISH. **a** Flow cell developed by Sieben et al. with straight channels in PDMS on top of a glass microscope slide. Reproduced by permission of the Institution of Engineering and Technology, Ref. [37]. **b** The commercialized microFIND^®^ device for microchannel-based FISH with (**i**) top view of channel design, (**ii**) side view showing the nanostructured titanium dioxide deposited atop a microscope glass surface. Reproduced with permission from Ref. [38]. **c** K562 cells after probe hybridizing in ‘FISHing line’ device, a narrow and shallow channel etched into a microscope glass slide, which allows trapping of cells into a confined space in string-like fashion for more straightforward visual inspection. Reproduced with permission from Ref. [41]. **d** Narrow inlet and outlet channels around deep chamber for cell trapping fabricated in COC. Reproduced with permission from Ref. [43]. **e** OncoCEE™ chip with a wide channel containing 9000 posts of varying diameter. The streptavidin-coated posts allow capture of biotin-tagged cells of interest. Figure adapted with permission from Ref. [44]. **f** Metaphase FISH with (**i**) splashing device featuring an open chamber for metaphase spreads preparation on a glass slide and (**ii**) PDMS flow cell to carry out FISH protocol of spread cells. Figure adapted with permission from Ref. [47]

The first article on FISH on-chip was published in 2007 by Sieben et al. [37]. They produced a glass device featuring ten parallel straight channels of 5-cm length flanked by a 1.5-µL well (Fig. 1a). The channels were 310 µm wide and 55 µm deep and sealed with a 170-µm thin cover plate. The team studied chromosomal abnormalities in peripheral blood mononuclear cells (PBMCs). About 15,000 cells were loaded into the inlet well and moved into the channel based on capillary forces. The chip was heated to 85 °C for 10 min to promote cell attachment on the channel surface. A vacuum was then applied to remove the remaining suspension, followed by introducing proteinase K, again via capillary forces. This process of loading and suction was repeated for the various permeabilization and cell treatment steps. Eventually 1 µL of probe solution was added, the inlet and outlet were blocked with a sealant, and the chip was left to incubate, with best results obtained for 4-h hybridization time. The team also investigated EOF for shunting liquids. Electrodes were placed into the reservoirs and 10 V/cm were periodically applied with pauses in between. Finally, the cells were washed and imaged through the thin cover plate. The physical confinement combined with a continual delivery of fresh targets by electrokinetic transport significantly reduced hybridization time and reagent consumption compared to conventional setups.

Zanardi et al. developed a microchannel system for FISH analysis with nanostructured titanium dioxide (ns-TiO_2_) (Fig. 1b) [38]. The 50-nm-thick TiO_2_ coating was deposited onto a microscope slide. Following chemical activation with plasma treatment, a slab of a soft polymer material (polydimethylsiloxane, PDMS) was bonded onto the slide. This PDMS slab featured a 10-mm-long microchannel of 300-µm width and 50-µm depth. This yielded a channel with an internal volume of 0.15 µL and a TiO_2_ surface area of 3 mm^2^. Samples were loaded over the ‘in’ well of 1.2-mm diameter and either left to move into the channel via capillary action or pulled through via a syringe with tubing connected to the 0.7-mm-diameter outlet. The device was initially tested with cultured hematopoietic tumor cells, bone marrow and peripheral blood. A 1.5-µL volume of cell suspension (10–20 × 10^3^ cells µL^−1^) was loaded into the device and allowed to attach to the titanium dioxide surface over a period of 4 min whilst applying 37 °C on a hotplate. The nanomaterial coating served to promote the trapping and thus immobilization of more than 1000 cells as they flowed through the channel. They stayed in place even under high shear stress conditions at aspiration rates of 5.5 µL s^−1^ (37 cm s^−1^). Liquid for the fixing, washing and probe hybridization was pumped by placing the desired volume over the inlet well and pulling with a syringe at the outlet. For the hybridization step, 0.3 µL of probe solution was introduced and left to incubate at 37 °C overnight. The final washing steps were performed in conventional dishes following removal of the PDMS slab. The spatially confined cells allowed for efficient imaging, around 200 cells were analyzed per sample. Due to its simple channel geometry, the device has potential for parallelization. This system has been commercialized under the brand name microFIND^®^ in combination with automated fluid handling and has been applied to genetic based cancer screening [39]. Ho et al. took on this system to carry out a prenatal analysis of chromosomal aneuploidies, requiring only 1 h for probe incubation and 3 h for the full protocol, coining the term ‘same day diagnosis’ [40].

Mughal et al. also employed a straight microchannel design for their FISH on-chip assay, however, with significantly smaller channel dimensions which they termed FISHing lines [41]. The 1-cm-long microfluidic channels, flanked by inlet and outlet wells of 1-mm diameter, were etched into glass microscope slides to a depth of 50 µm with a width of about 45 µm at the top and 30 µm at the bottom of the channel. The channel dimensions, thus, marginally exceeded the dimensions of K567 cells (35 µm) and Jurkat cells (25 µm), which were employed here for minimal residual disease (MRD) analysis in leukemia. Initially, the microchannel plate was used without lid. The cells were fixed off-chip, and a 0.2-µL volume of cell suspension (2 × 10^3^ cells µL^−1^) was pipetted over the channel and allowed to air dry; the cells were, thus, attached to the channel surface. The channel was then covered with adhesive film and 0.2 µL of probe solution was pipetted over the inlet and moved through by gentle manual suction with a syringe at the outlet. The inlet and outlet were then sealed during denaturation (5 min at 75 °C) and hybridization (2 h at 37 °C). After this, the adhesive cover was removed, and the glass slide with the now open channel was washed as conventionally. Fresh adhesive was then applied to perform a final step of DAPI staining. The relatively close match of channel size and cell size resulted in cells being aligned in a string-like fashion (Fig. 1c), which allowed relatively convenient visualization; ten channels fitted onto a single microscope slide for parallelization. On the other hand, the small channel size and small sample volumes employed limit the number of cells that can be deposited, 400 cells per channel compared to 50,000 on a conventional flat microscope slide.

Descroix’s team has pursued a different cell loading strategy, narrow microchannels (60 µm wide, 30 µm deep) leading into and out of a deep round chamber (380-µm height, 1-mm diameter at bottom) (Fig. 1d) [42, 43]. Cells flowed fast through the narrow microchannels but slowed down and settled within the deep chamber. The device was hot embossed in cyclo olefin copolymer (COC) and closed with a 145-µm thin COC film. This process and material are amenable to mass fabrication whilst still allowing sufficient optical transparency for microscopy. The device was interfaced with a programmable pressure-based fluid handling system which allowed automatic injection of all reagents. The COC surface was pretreated with cellulose and lysine solutions to promote cell adhesion. All reaction steps were performed inside the channel system, without any drying steps or opening the device. The authors found they could dilute the probe concentration up to a factor 4 on account of the efficient perfusion in the microchannel and avoidance of the drying step. The chip was applied to quantify ERBB2 gene amplification; an ERBB2-amplified and unamplified cell line were studied as well as two clinical samples. The performance matched the gold standard microscope slide test, whilst reducing sample volume, assay times and increasing automation.

The concept of a widening chamber was also applied in a system for Cell Enrichment and Extraction (CEE) of cancer cells, termed OncoCEE™, developed by Biocept *Inc.* [44–46]. A microfluidic structure was fabricated in PDMS and mounted on a thin glass coverslip to enable high-resolution fluorescence microscopy at 200× total magnification. The design featured a branched channel inlet network leading to a 12-mm-wide, 40-mm-long and 55-µm-deep chamber with around 9000 posts of variable diameters between 75 and 150 µm. These were engineered to disrupt regular streamline flow and maximize the probability of contact between cells and the large surface area of the posts (Fig. 1e). The minimum post distance was set to 70 µm to avoid clogging with cell clumps, with the posts occupying about 25% of the chamber which could hold 15 µL of fluid [44]. The surface of the posts was modified with streptavidin. Mayer et al. [45] and Krishnamurthy et al. [46] applied this system for FISH-based detection of HER2 status of circulating tumor cells (CTCs) in peripheral blood and also bone marrow of breast cancer patients. Following tumor cell enrichment, a range of biotinylated antibodies against proteins expressed on the surface of CTCs were added to the blood or bone marrow samples. When these were pumped through the post array, the now biotinylated CTCs could be captured on the posts directly from the blood sample. Cells were initially stained with fluorescently labeled antibodies to allow counting and location determination. Reagents for the HER2 FISH assay and DAPI staining were then pumped through the device using the group’s custom-designed high-precision pumping system.

Vedarethinam et al. [47] developed a microfluidic device to perform FISH in the metaphase of the cell cycle, which allows the study of insertions, deletions or rearrangements of specific regions within the genome. This requires careful handling and fixing of chromosomes to obtain a consistent and high-quality metaphase spread. The authors studied peripheral blood lymphocyte cultures to visualize X chromosomes. A conventional microscope glass slide was employed as substrate that could be interfaced initially with an open chamber PMMA splashing device (Fig. 1fi) to deposit the metaphase spread and then with a closed PDMS flow cell (Fig. 1fii) to perform the FISH assay. For the splashing device, a 50-µm-thick double-sided adhesive tape was laser-cut to reveal a rectangular channel. Initially, only one side of the adhesive cover was removed to fix the tape on the microscope slide. The slide was then fitted into a poly(methyl methacrylate) (PMMA) assembly holding it in position below an open chamber. Tubing was inserted to constitute a splashing device with two inlets for cold water and cell suspension, respectively, and an 11-mm dropping height. This allowed for controlled evaporation of the fixative leading to stretching of the chromosomes and flattening of the cells, a key step in the preparation of high-quality metaphase spreads. Following spreading, the glass slide was removed from the PMMA assembly and the top cover of the double-sided tape was also removed to allow attachment of a PDMS flow cell. RNAse, washing and drying solutions were pumped through the flow cell over the fixed cells, a hotplate was used for heating as required. For probe hybridization, the flow was stopped, the inlet and outlets were closed, and the device was left to incubate overnight. The low dead volumes in the microfluidic system allowed for a 20-fold reduction in the total reagent volume consumed. It is argued that non-technical personnel can perform the interchange from splashing device to FISH flow cell rapidly. The group further developed their devices to integrate cell expansion, required as a pretreatment for metaphase chromosome spreads from lymphocytes [48, 49].

### Trapping cells on membranes or in regular arrays

Several research teams have opted for trapping cells in regular arrays to improve control of the cell microenvironment and the automation of imaging of up to thousands of cells at the single cell level. Liu et al. developed a PDMS stamping method to pattern a microarray of cell anchor points onto a glass slide, FISH assay steps were conducted by pipetting reagents into a well (13-mm diameter, 3-mm high) atop the array or via dipping the slide into solutions [50]. Lee et al. fabricated a 16 × 6 array of PDMS wells, each of 1.5 mm diameter, onto a gold functionalized glass slide, which was utilized for cell attachment and then removed to carry out the FISH assay in a conventional manner [51]. Neither of these systems featured microfluidic flow channels.

Matsunaga et al. developed a microfluidic device with PDMS channels sandwiched around an array of 10 × 10 microcavities of 2 µm diameter that were laser ablated into a 38-µm-thick sheet of black PET [52]. The PDMS flow cells featured a top channel with sample inlet and outlet and a circular section over the micromesh as well as a bottom channel which ran from below the micromesh to an outlet connected to a vacuum pump. Mammalian Raji cells were fixed off-chip and a 5-µL suspension containing about 50 cells was pulled through the device by negative pressure. The cells were found to be trapped uniformly over the micromesh, non-specific adsorption to the PDMS was minimized with plasma and Pluornic F-127 treatment. Permeabilization was achieved by incubation with 50% ethanol at 60 °C on a hotplate. A solution of red fluorescent oligonucleotide probes targeting β-actin mRNA was then loaded into the device, inlets and outlets were sealed, the device was placed in a humidified chamber and left to incubate at 42 °C for 2 h. Next, excess probes were thoroughly washed out and cells were visualized under the microscope. The team showed proof-of-concept data for different expression levels in cells either supplied with serum or starved of serum.

Kurz et al. fabricated a 35 × 35 microhole-array in silicon. The holes were 5 µm in diameter and arranged 60 µm apart. Following demonstration of cell trapping [53] the device was applied for FISH analysis [54] of human retina pigment epithelia (ARPE-19) cells. The cells were loaded via negative pressure and left to attach for 3 h before the FISH solutions were pumped through the device. The hybridization was carried for 14–20 h. The authors reduced the assay reagent consumption by a factor of 5 compared to the standard protocol and also developed a software for automated image analysis.

**Fig. 2:**
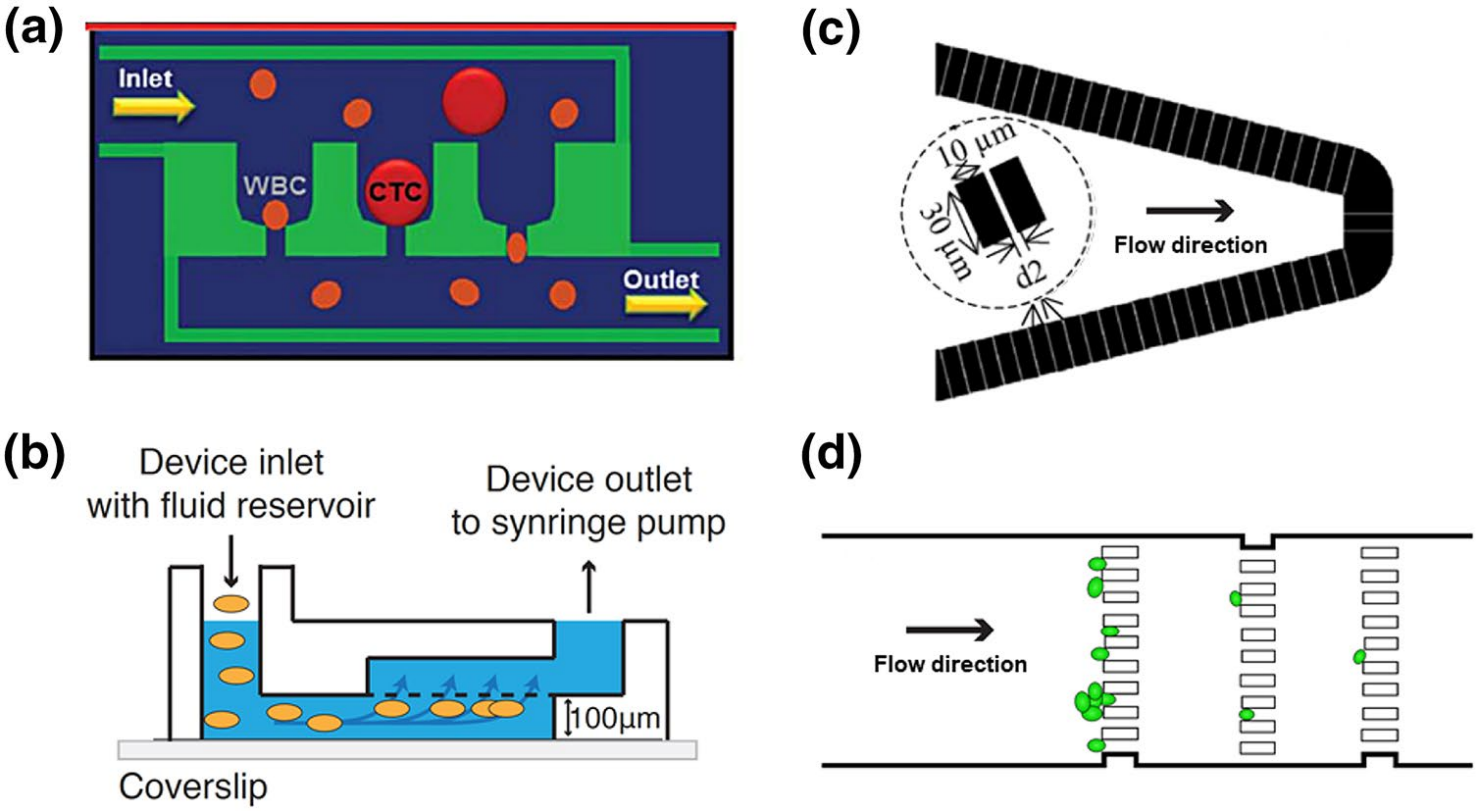
Microfluidic cell trapping systems for FISH. **a** Concept of Celsee microfluidic chip for capture of CTCs in microfluidic traps with a pore channel allowing smaller and deformable red blood cells and white blood cells to pass. Reproduced with permission from Ref. [56]. **b** Concept of microfluidic device for trapping of virus infected MDCK cells at the bottom of a 5-µm pore size membrane. Reproduced with permission from Ref. [57]. **c** In-line flow through device with microfabricated pillars in silicon, featuring 1–2-µm gaps between weirs for capturing of *Giardia lamblia* cells. Reproduced with permission from Ref. [58]. **d** In-line flow through device for trapping yeast cells with rows of microfabricated pillars in PDMS with 5-µm gaps. Reproduced with permission from Ref. [59]

Riahi et al. developed a cell capture device which allowed trapping of cells in > 56 k  individual capture chambers (20 µm side length, 30 µm depth) with a pore channel at the bottom of 7.5 µm width (Fig. 2a) [55]. Cell suspensions were passed through a 75-µm-deep fluidic network leading to the cell trapping chambers. Small or deformable cells such as red blood cells (RBCs) and most leukocytes could pass through the pores and were flushed out in an outlet channel system below, whilst larger CTCs were trapped. This device has been commercialized as Celsee PREP 400™ system and includes automated sample delivery whereby reagents are dispensed into an inlet funnel and pulled through the device by vacuum, as well as an automated image readout. Gogoi et al. [56] applied this technology to enrich CTCs from blood samples of patients with metastatic breast, prostate and colorectal cancers and carried out an on-chip DNA FISH assay for HER2 as well as a FISH assay for mRNA expression. In these cases, probes were incubated at 37 °C overnight. The Celsee system was shown to have a higher sensitivity than the gold standard method (CellSearch).

Shaffer et al. also employed a membrane with microcavities [57], in this case, a circular piece of a commercially available polycarbonate track-etched film featuring a pore diameter of 5 µm. Microchannels were laser-cut into 100-µm-thick double-sided tape. The bottom was fixed to a microscope cover slip (150-µm thick) to allow high-quality fluorescence imaging, the top was stuck onto a piece of acrylic with inlet and outlet holes (Fig. 2b). Liquid was pipetted into an inlet reservoir mounted on top. Cells were guided to the membrane from the liquid reservoir through the lower channel by negative pressure at the outlet via syringe pump. The cells were, thus, pushed against the membrane, where they got entrapped. The group studied viral infection based on viral RNA FISH analysis, specifically looking at influenza infected Madin-Darby canine kidney (MDCK) cells. An RNA probe mixture featuring 20–50 short probes was custom-designed to distinguish between viral subtypes. Following fixing off-chip, around 1000 cells were loaded into the device which was placed on a hotplate held at 37 °C. After a wash, the probe mixture was introduced and left to incubate, repeatedly, in total 40 µL of probe solution was used over a period of only 5 min. The short incubation time was enabled by high probe concentrations and the efficient perfusion in the microfluidic flow cell. Following a range of washing steps, fluorescence microscopy was carried out. The pump protocol could be automated; the user was required to pipette solutions into the inlet well. Image analysis was also automated. The entire FISH assay and imaging required only 15 min.

Zhang et al. demonstrated an in-line weir flow through system for trapping *Giardia lamblia* cells (7–10-µm wide, 8–13-µm long) (Fig. 2c) [58], employing a silicon base plate and 500-µm-thick glass cover plate. The channel design was etched into the silicon base to a depth of 50 µm with a channel of several mm width. Liquid initially encountered a region with several rows of coarse filter posts, *i.e.,* 30-µm-wide obstacles spaced a few tens of µm. Further downstream, the liquid passed through a weir structure, each weir 30-µm long and 10-µm wide with a gap of 1–2 µm between weirs. The device was placed on a heating plate and interfaced to a presume pump via tubing. Cell suspension was pumped through and cells became trapped. Probe solution, which was diluted by a factor 10, was pumped and best results were obtained at 1 µL min^−1^ with a pumping time of about 10 min. While this type of precise microfabrication is relatively involved and costly, it does allow trapping of relatively small cells.

Ferreira et al. devised a flow through channel with microfabricated in-line pillars serving as obstacles to trap yeast cells and carry out a FISH assay with peptide nucleic acid (PNA) probes targeting rRNA [59]. These relatively small and non-charged probes allow for faster hybridization times. The devices were fabricated from PDMS and a range of channel and obstacle geometries were investigated computationally and experimentally. The design performing best in terms of trapping efficiency featured a straight channel of 100-µm width and 30-µm depth with three rows of 15 µm × 45 µm pillars with 5-µm gaps between them (Fig. 2d). Liquids were pipetted over an inlet reservoir and pulled through the device by applying negative pressure at the outlet. Yeast cells were fixed off-chip and about 50,000 cells were pumped into the device. The hybridization step was carried out for 60 min at 59 °C, followed by extensive washing and fluorescence microscopy.

Ismagilov’s team used droplet microfluidics [60] to confine a population of bacterial cells, *Paenibacillus curdlanolyticus,* into individual nanoliter-sized droplet plugs [61]. These were merged with droplets containing ethanol for the fixation and incubated at − 20 °C for 20 h. The droplets were then spotted into a microwell and the remainder of the FISH protocol was carried out in the wells by pipetting the relevant solutions. The hybridization step was performed with 100-µL probe solution for 2.5 h at 48 °C.

Packard et al. used dielectrophoretic (DEP) forces to trap bacterial cells in microchannels, followed by a fluorescent-resonance-energy-transfer-assisted ISH assay (FRET-ISH) [62]. They fabricated interdigitated Cr–Au metal electrodes of 40-µm width, 40-µm spacing and 250-nm thickness onto a silicon wafer bonded to a glass slide with a channel of 60-mm length, 2.6-mm width and 10–15-µm height. Liquid was introduced through a syringe pump usually at 100 µL min^−1^ (around 50 mm s^−1^). Temperature was controlled by Kapton heater adjacent to the chip. Cells were trapped over a period of 1 min followed by introduction of the various solutions for permeabilization and probe hybridization. Samples were heated at 65 °C for 5 min for denaturation followed by incubation at 25 °C to allow probe hybridization, which was detected in less than 30 min.

The published trapping devices range from commercially available filter membranes to high-end clean room fabricated trapping systems. The filter pore size or gaps between pillars are dictated by the desired cell sizes, with larger yeast cells or CTCs easier to trap than smaller bacterial cells. Employing DEP forces for trapping avoids a pillar or filter system but does necessitate the microfabrication of electrodes.

### Integrated and automated devices

Several research groups have set out to integrate all the FISH assay steps into one monolithic device, a ‘sample-in-answer-out’ system to perform the entire protocol in a sequential and automated manner. The vision here would be to have a bench-top control box with the lab-on-a-chip device as a disposable item. Whilst this is attractive in terms of market penetration, it is also very challenging, given the number of reaction and washing steps, the range of solvents and the temperature control required.

**Fig. 3:**
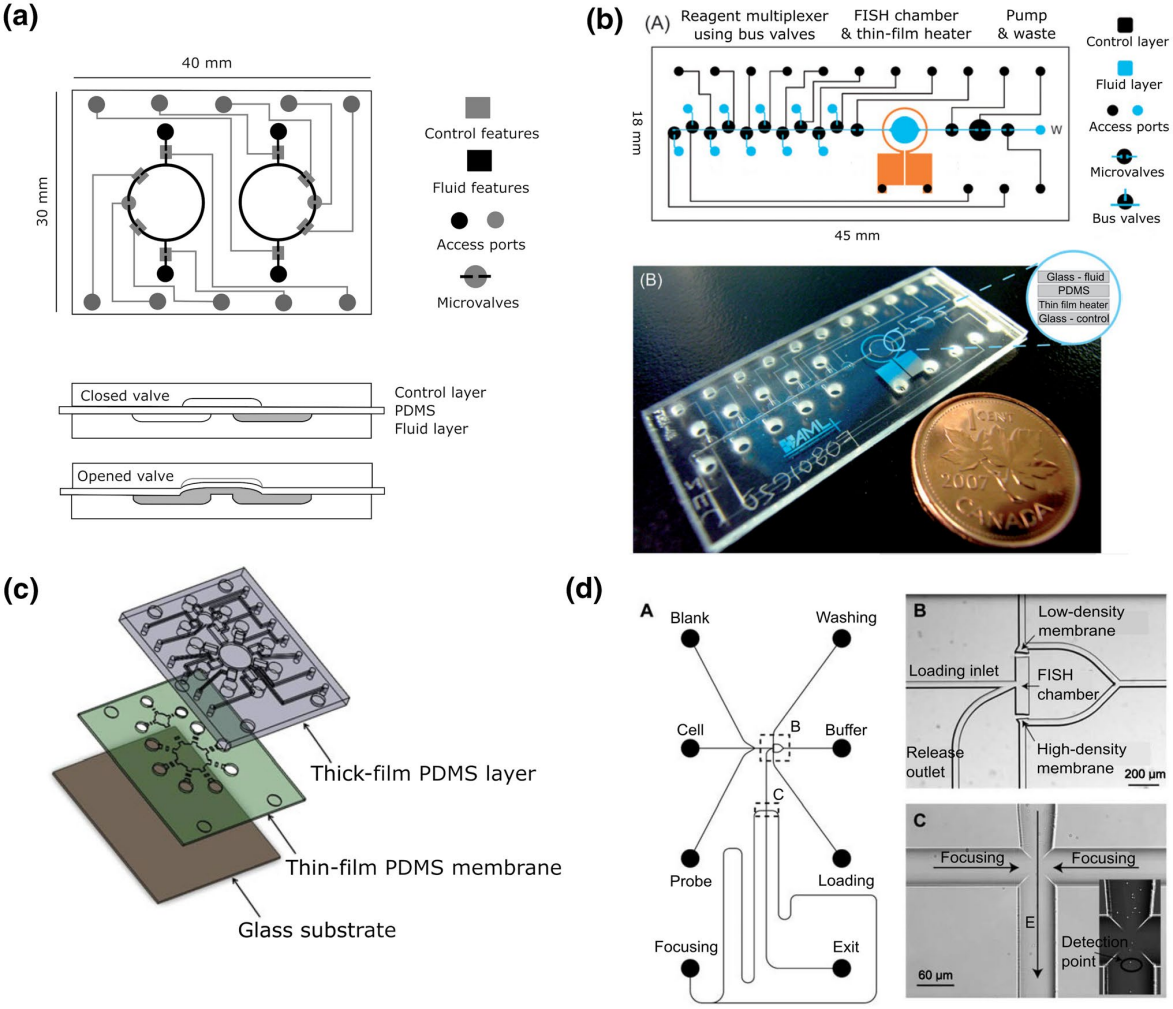
Integrated FISH on-chip systems. **a** Top view and cross section of the circulating microchip with PDMS valves for fluid actuation. Reproduced by permission of the Institution of Engineering and Technology, Ref. [37]. **b** Conceptual drawing and photograph of fully integrated FISH assay device with on-board valves and heater featuring microchannels in glass in the top and bottom layers and a thin PDMS layer to actuate the valves. The device had ten wells for reagents and one waste outlet. Reproduced with permission from Ref. [56]. **c** Three-layer chip devised by Lee’s group with a rigid, flat glass bottom layer, a thin fluid-carrying PDMS layer and a thick PDMS layer with air-carrying channels to actuate valves. The system featured a reaction chamber, nine reagent reservoirs and a waste outlet. Reproduced with permission from Ref. [65]. **d** µFlowFISH system combining the FISH assay with downstream cell focussing and flow cytometry readout. Pumping was achieved via EOF. Gel plugs of different porosities acted as filters to retain cells and probes but let smaller molecules pass. Reproduced with permission from Ref. [66]

Perhaps surprisingly, the first publications on microfluidics-based FISH by Sieben et al. in 2007–2008 [37, 63] featured a very high level of integration and automation. They presented a device with a circular channel to allow recirculation of probe over immobilized cells (Fig. 3a) based on pneumatic pumps and valves inspired by the work from Mathies’ group [64]. The device featured a rigid bottom layer with channels of 580-µm width and 40-µm depth etched into glass. This included a circular channel of 5-mm radius and two straight channels at opposite sites leading to 1.5-µL wells. A flexible middle layer was made from 0.25-mm-thick PDMS which could be deformed to open and close the channel beneath. A rigid glass top layer featured control channels filled with gas through which positive or negative pressure could be applied to deform the PDMS. This on-chip actuation system reduces dead volumes associated with external tubing. Temperature control was achieved by mounting the device onto a thermocycler. The team studied chromosomal abnormalities in PBMCs. Cells were loaded into the device and adherence was enhanced by heat treatment. Solutions for digestion, dehydration and fixation were introduced sequentially. Eventually, 1 µL of probe solution was loaded into the device and circulated at one cycle per minute by the actuation valves. Best results were obtained with 4-h hybridization time.

A year later, a fully integrated and automated device was presented for chromosome enumeration [63] (Fig. 3b). The device consisted of three layers: a rigid top layer made from glass with fluid-carrying channels, a thin flexible middle layer of PDMS was again used for actuation, and a rigid glass bottom layer with pressure control channels for 14 valves. The device featured no fewer than ten reagent reservoirs along a central straight channel, 150-µm wide and 50-µm deep, leading to a circular FISH chamber (2.5-mm diameter, 250-nL volume) and a waste outlet. A viewing port was required beneath the FISH chamber to enable fluorescence microscopy. The device also included a thin-film platinum heating element between the PDMS and bottom layer. The entire setup with fluid and valve control and thermocouples for temperature measurements could be mounted on a microscope stage to allow for real-time imaging. The user would be required to load reagents into the respective wells and the 1.5-h protocol then ran completely automatically by suction from a peristaltic pump and opening and closing valves as required to perform cell loading, digestion, dehydration and fixation, washing and drying steps, as well as probe hybridization and DAPI staining. Probe volume used was in the 0.5–1 µL range and was left to hybridize for 60 min at 37 °C. The device was again applied to PBMC cells including patient samples for analysis of X- and Y-chromosomes per cell in the context of chromosomal abnormalities.

Gwo Bin Lee’s group further developed the concept of fully integrated, automated stand-alone FISH-on-chip systems [65]. Their three-layer device also featured a flexible PDMS middle layer that could be deformed to open or close channels. However, no glass microfabrication was used here; instead, the fluid-carrying channels were cast in the thin PDMS slab, which was placed on top of a flat glass slide (Fig. 3c). The fluidic layout included a pump chamber, a circular reaction chamber of 4-mm diameter and 200-µm depth, a channel network leading to nine reagent reservoirs and one waste outlet. A rather thick slab of PDMS was employed as top layer, featuring air-filled channels through which positive or negative pressure could be applied to open and close valves and actuate the lids of two circular chambers for fluid transportation or mixing. The device was placed on top of two temperature control blocks, the area below seven of the storage reservoirs was kept at 25 °C to prevent reagent degradation, whilst the temperature in the area below reaction chamber and two of the reagent reservoirs was varied between 37 and 73 °C as required for the various steps. Reagents for cell fixation, hybridization and post-hybridization treatment could be preloaded into the nine reservoirs. Cell suspension mixed with fixative was directly loaded into the reaction chamber and the various assay steps were run automatically with liquid volumes ranging between 10 and 300 µL. The hybridization reagent comprised of 0.5 µL probe solution diluted to 5 µL with buffer. Pressure was applied to push down the ceiling of the reaction chamber, thus reducing its height to around 50 µm and reducing the required diffusion distances for probe hybridization. This, together with gentle actuation of the lid, meant the hybridization time could be reduced from 16 h in the conventional setup to only 40 min on-chip. For imaging, the device was transferred onto a microscope stage. The team demonstrated the feasibility of this approach by assaying for chromosomal abnormalities in leukemia cells. The device was further developed to study HER2 expression in cell and tissue samples (see next section).

Whilst PDMS valves and actuators are elegant in nature, they require an external pressure control unit and a significant number of connectors on the device to operate the various ports. Fluid control via electroosmotic flow (EOF) offers an alternative. For this, electrodes are dipped into channel reservoirs, an electric field is applied, and bulk liquid movement is induced. The direction and speed of this are determined by the applied electric field. Liu et al. developed a combination of FISH and downstream flow cytometry readout, termed µFlowFISH [66] (Fig. 3d), that makes use of this strategy for the identification of bacteria in microbial communities. FISH was used to label 16S rRNA in bacterial cells, followed by cell focusing and flow cytometric detection. This integrated approach allows tracking of individual bacteria, enables further molecular analysis and ensures labelling and detection happening on the same volume scale, minimizing sample losses. The device was entirely fabricated from glass to support EOF pumping. At its core it featured a FISH chamber (120-µm wide, 20-µm deep) with three access points and a channel network leading to eight reservoirs for reagents and waste. On two of these FISH chamber access points, different porosity plugs of polyacrylamide gel were generated through photopolymerization. These acted as size selective filters, retaining cells and probes but allowing small molecules to pass freely. The device was placed onto a heat-controlled microscope stage and connected to electrodes for EOF. Cells were fixed off-chip and introduced into the FISH chamber. EOF was then used to pump reagents from their respective reservoirs through the FISH chamber. The system required 80 µL of probe solution. An alternating electric field was used to shunt probe along the FISH chamber in six 5-min cycles at 46 °C to enhance probe and cell interaction. Finally, the cells were pumped towards a channel cross section and focused by two sheath liquids into a narrow stream for laser-based flow cytometry readout of fluorescence and scattering on the same chip.

## FISH with tissue samples

The reports on microfluidic approaches to FISH analysis listed so far have focused on analysis of cells in suspension or cells immobilized in layers. In a clinical context, it is often of interest to study a tissue slice, such as paraffin-embedded biopsies. These are typically several mm in side length or diameter and a few µm thick. For utilizing these within microfluidic systems, it is important to place and maintain an integral slice into the device and achieve appropriate perfusion of the tissue with reagents. The thicker the tissue, the more time will be required, the larger the volume of probe solution and the higher the dangers of non-uniformity. Finally, the tissue needs to be imaged over a sufficient field of view with sufficient spatial resolution and consideration needs to be given to the thickness and optical quality of the chip material above and below the tissue.

### Tissue slices in fluidic chambers

**Fig. 4:**
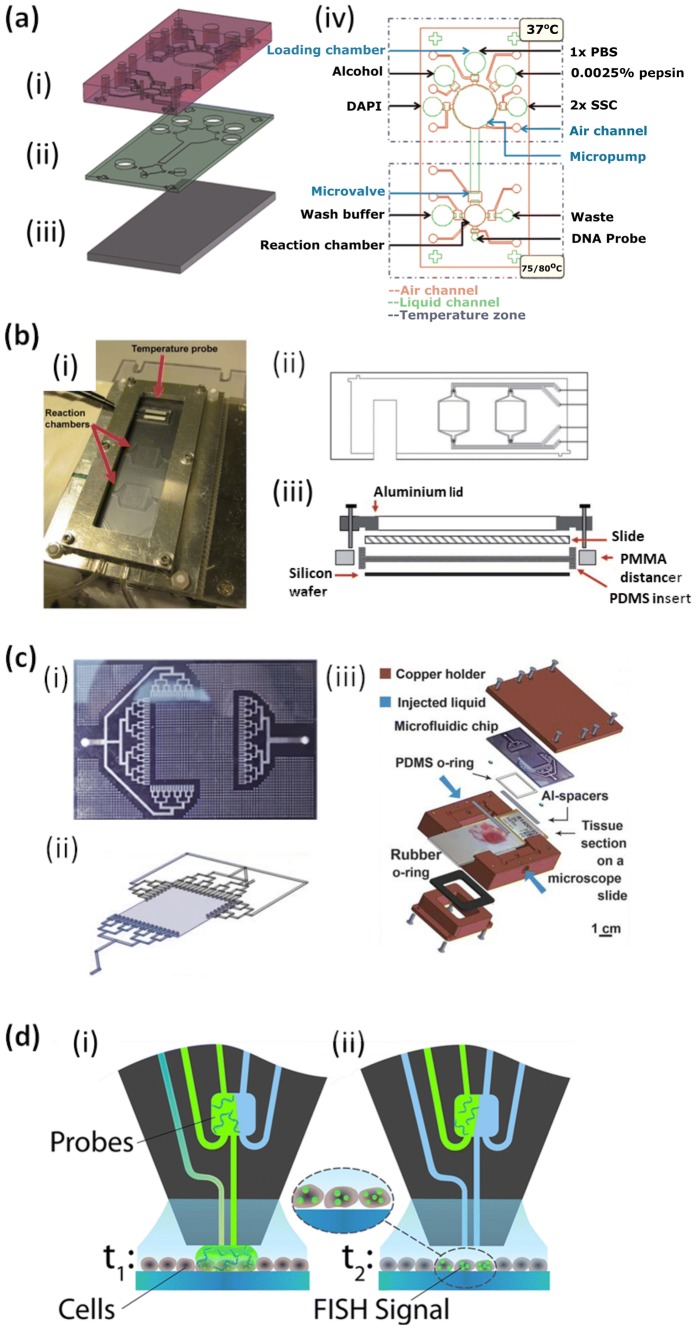
Microfluidic devices for FISH assays on tissue slices. **a** Exploded view of Lee group device composed of (**i**) an air layer, (**ii**) a liquid chamber layer, and (**iii**) a glass slide. (**iv**) Top view drawing of fluidic network over two temperature zones. The device housed a paraffin-embedded gastric cancer biopsy slice for study of HER2 expression. Reproduced with permission from Ref. [67]. (**b**) (**i**) Photo, (**ii**) top view and (**iii**) side view of the HistoFlex device with a PDMS flow chamber of 10 mm × 10 mm × 100 µm on a silicon wafer with microscope glass slide lid. The device housed brain tissue slices for analysis of 18S rRNA and miRNA. Reproduced with permission from Ref. [68]. **c** The MA-FISH device developed by the Gijs group with a branched channel structure delivering and withdrawing fluid to and from all sides of tissue slice. (**i**) Photograph of fluid-carrying glass layer allowing a homogenous distribution of the liquid for uniform staining of the tissue slice. (**ii**) Conceptual drawing of tissue chamber and microchannels, (**iii**) exploded view of full device featuring microscope slide with tissue, Al-spacers and PDMS O-ring to create a 20-µm-high tissue chamber with fluid delivered through the glass slide layer with etched channels. The device was tested for HER2 expression in 4-µm-thick cancer tissue slices. Reproduced with permission from Ref. [69]. **d** Concept of vertical microfluidic device with inlets and outlets for confined delivery and withdrawal of nL volumes of (**i**) hybridization probes and (**ii**) wash buffer. Reproduced with permission from Ref. [77]

The fully integrated device presented by Tai et al. to study of chromosomal translocation in cell lines (Fig. 3c) [65] was further developed to enable analysis of parafilm-embedded tissue slices for HER2 expression (Fig. 4a) [67]. A glass bottom layer was clamped together with two PDMS layers. The PDMS layers could be removed following the FISH assay to enable high-quality microscopy readout. The fluidic network included a 5-mm-diameter reaction chamber and a 10-mm-diameter pumping chamber, which could be actuated to transport liquid between the reagent reservoirs, the reaction chamber and the waste port. The reaction chamber could house a 5 mm × 5 mm and 2.5-µm-thick paraffin-embedded tissue slice from breast cancer biopsies which was loaded before chip assembly. The team studied HER2 expression with the hybridization being carried out at 37 °C over 16 h. The device was found to yield comparable results to the conventional method, but with much reduced assay time and reagent volume consumption and in a largely automated manner.

The Dufva group developed a flow cell for holding histological tissue sections, termed HistoFlex [68]. The device comprised of a silicon waver, a PDMS channel layer and a conventional microscope slide as top layer (Fig. 4b). It was housed in a frame made from aluminum and PMMA and placed on a custom-designed temperature control unit. Fluid was delivered through a network of tubing and valves with eight inputs and one output to enable user-friendly and bubble-free interchange of input liquids. The core part was a flow chamber of 10 mm × 10 mm × 100 µm housing the tissue slice. A triangular widening section of 400-µm depth was found to deliver liquid fairly equally across the area of the tissue. Formalin-fixed and paraffin-embedded tissue was sliced at 4-µm thickness and was prepared off-chip, including dewaxing, rehydration and fixation and pre-incubation at 37 °C before placing into the *HistoFlex* device. Hybridization probe was recirculated at 20 µL min^−1^ for 15 or 60 min at 45–50 °C. The team studied 18S rRNA and miRNAs in mouse brain tissue sections. The effective liquid delivery allowed the overall protocol to be performed in about half the normal time, *i.e.,* 3 h. The device was reported to perform with significantly improved sensitivity, achieving a high degree of hybridization uniformity across the reaction chamber and low slide to slide variation.

The Gijs group developed a flow cell for tissue perfusion to study HER2 expression [69] termed microfluidics-assisted FISH (MA-FISH). Here, the tissue slice was mounted on a conventional microscope slide and placed into a custom-made copper holder. A PDMS ‘o-ring’ and aluminum strips were placed on the glass slide to form a 16 mm × 16 mm chamber of 20-µm height around the tissue slice. On top of this, a fluidic network, etched in glass, was assembled (Fig. 4c). The network was comprised of branching channels that delivered and withdrew liquid to and from all four sides of the tissue section. Indeed, the team applied ‘square wave oscillatory flow cycles’ to ensure best possible perfusion during their FISH assays. Fluid flow was controlled with individual channel syringe pumps. Temperature was controlled though an external hotplate. Samples were obtained from a biobank of breast cancer tissues, cut into 4-µm-thick slices and underwent de-paraffinization, pre-treatment, protein digestion, post-fixation and washing before the chip was fully assembled. For the time-limiting hybridization step, 10 µL of diluted probe solution was loaded into a syringe and push-pulled through the tissue containing chamber with 5-µL displacement volume over a period of 4 h. This enabled efficient delivery of the probes to the tissue. For image analysis, the microscope slide was removed from the holder and placed on the microscope stage. The entire protocol could be performed in a working day compared to several working days required for the standard protocol. In a follow-up paper [70] the device was used to compare HER2 FISH on cell and tissue samples with chromogenic in situ hybridization (CISH), which enabled bright-field rather than fluorescence readout. Furthermore, the device was adapted for extra-short incubation microfluidics-assisted fluorescence in situ hybridization (ESIMA-FISH) [71], using a highly reactive probe mixture and optimizing the assay protocol. Thus, the hybridization time could be reduced to 15 min for cells and 35 min for tissue slices. In a further article, the team demonstrated the study of intra-tumoral heterogeneity with sequential immunofluorescence assay and FISH staining on the same tissue section combined with automated image processing [72].

### Vertical microfluidic probe for cell layers and tissue slices

Kaigala’s team has developed a rather different concept to performing FISH on tissue slices, a non-contact vertical probe that delivers liquids to a small area of the tissue and can be moved to scan over the sample. Here, the tissue slice in not enclosed in a flow cell, but instead open to the environment. A tip with two inlet and two outlet microchannels (Fig. 4d) can deliver fluids to the tissue surface and pull liquids away. The probe head was made from silica and glass with six channels, two for injection, two for aspiration and a further two outer channels to replenish the immersion liquid on the substrate without direct interaction. The channels were etched to a depth of 100 µm, coming to the apex at 100 µm × 100 µm for the innermost channels, 50 µm apart from each other, and 100 µm × 200 µm for the washing channels. A glass slide with the sample mounted was placed on a microscope stage, the fluidic probe was fixed to a linear stage for movement over the sample at about 20-µm distance from the sample. The probe head was interfaced with a syringe pump system. This allowed for manipulation of nL volumes and interrogation of an area of 300 µm × 300 µm [73, 74], with low dead volumes and very short diffusion distances down to a few µm. The hybridization probe solution could also be switched over so that different areas of the sample could be interrogated with different probes. Kaigala’s team applied this to the rate limiting step in FISH assays, *i.e.,* having the device deliver the hybridization probe followed by a washing step. The device can essentially scan over a cell layer or tissue slice, measuring in zones of interest in a fast manner. The probe was first applied to FISH on breast cancer cell lines with about 1000 cells interrogated at a time [75]. Signals were obtained with hybridization times as short as 3 min with 0.6 µL of probe, followed by a 2-min wash, faster than any other method reported for FISH. In a follow-on paper [76], HER2 and Cen17 were studied in parafilm-embedded breast tissue sections. With the 300 µm × 300 µm probing area, around 300 cells were analyzed at once, which is often sufficient for a cancer scan. For the Cen17 probe, hybridization times as short as 1 min were  reported; whereas the HER2 probe required 15 min to yield a signal. Probes could also be shunted over the tissue to reduce their required volume per test to around 100 nL. Indeed, with the probe hybridization so fast, the team was also able to study the kinetics of probe hybridization [77].

## Comparison of microfluidic FISH platforms

A fairly wide range of design and engineering approaches are available to trap and immobilize the cells and tissue sections, introduce FISH reagents, control temperature and carry out the fluorescence microscopy readout. The diversity of microfluidic devices for FISH assays on cell suspensions, cell layers and tissue slices are summarized in Table 1.

There is no standardization in the peripheric instrumentation either, a variety of methods for pumping, interfacing to pumps and heating are reported. Many of the devices have been fabricated from glass, due to its favorable optical properties, or from PDMS, which is a preferred material for prototyping in many research laboratories. Many of the devices, however, would be rather expensive, in some cases prohibitively expensive to mass fabricate. One team addressed this issue and investigated the suitability of cyclo olefin copolymer (COC), which can be injection molded [43].

Major differences are also seen in the level of integration of the procedures. Several of the cell and tissue preparation steps are carried out off-chip and some of the devices need to be disassembled for microscope readout. The probe hybridization, however, is always done on the chip device. Some groups have opted to make their FISH devices fit seamlessly into the general laboratory workflow with microscope slides as substrates or using equipment generally available in laboratories such as hotplates. Loading reagents by pipetting also appears an acceptable option. Others have aimed at fully integrated standalone devices. The idea is certainly intriguing, but the reality is that the flow cells and setups of these integrated systems may be too complex to manufacture and run cost-effectively at larger scales. In addition, these closed designs most probably compromise the use of the systems in other, even similar, applications.

Samples under study have included mammalian cells in the interphase and metaphase of the cell cycle, pathogen cells as well as tissue sections. The different applications of conventional FISH, whether gene mapping, diagnosis of chromosomal aberrations and identification of pathogens, have all been shown to work on lab-on-a-chip devices, with the HER2 assay being the most popular case study (see Table 1) among the assays shown on-chip. Depending on the device layout and application, the number of cells under investigation varied, some using only 50–100 cells [41, 52, 58] or on the order of 1000 cells [57, 75], others at tens of thousands [56, 59].

The devices are generally reported to perform well, comparable to standard assays. However, the published articles only demonstrate initial proof-of-concept studies. Full characterization and analytical, let alone clinical, validation is lacking. For wider uptake, the devices have to be presented to regulators with much more data than currently published. Commercialization has been pursued for some of the approaches, such as the microFIND device with a straight channel for cell trapping on the nanostructure surface and the Celsee device with cells being trapped in a regular array with holes.

## Conclusion

The diverse microfluidic platforms described in the literature so far have addressed, to some extent, the key challenges of conventional FISH protocols, namely, improving on the long time to result, the labor-intensive procedures, the lack of automation, as well as the relatively high cost of reagents, especially the hybridization probes. Future development of FISH platforms will probably evolve towards further integration of the FISH procedure, which has not yet been fully achieved for many of them. Also, efficient trapping of small size cells such as bacteria from complex biological samples, remains challenging considering the limited number of studies addressing this subject and the fact that most studies use laboratory cell suspensions for testing that do not mimic the challenges of real biological samples.

Building on recent advances in FISH techniques, the field is now poised to evolve into novel directions, which in turn impose a new set of challenges. Examples for significantly enhanced performance of FISH on-chip platforms might include: (1) single cell analysis, (2) the target of low copy nucleic acid sequences or (3) highly efficient multiplex approaches. (1) Single cell analysis for studying cell-to-cell variability requires not only an efficient trapping system able to evaluate a relatively high number of individual cells, but also a powerful optical system for recording data which, so far, has not been integrated into the microfluidic platforms. The complexity of such trapping and optical systems will increase as the cell size decreases. This will require a multidisciplinary and complementary approach beyond the capability of single research groups. (2) To assess information encoded into low copy number nucleic acid sequences of small cells, such as the chromosomal DNA of bacteria, signal amplification has to be incorporated into the microfluidic system. Examples of FISH techniques resorting to signal amplification to access low copy sequences include Catalyzed Reporter Deposition-FISH (CARD-FISH) [78] or Recognition of Individual Genes-FISH (RING-FISH) [79] which have not yet been integrated into microfluidic devices. (3) Multiplexing in FISH techniques has been limited to the number of color channels available with the fluorescence microscopes, i.e., usually three color channels. Techniques based on spectral imaging have been developed to significantly increase the number of simultaneous targets. Combinatorial Labeling and Spectral Imaging (CLASI)-FISH can discriminate more than 20 targets simultaneously [80]. For integrating such techniques, the optical system must include spectral detectors. Dedicated software for imaging analysis is also needed in such a device. The fact is that only a few laboratories worldwide have the ability and the costly equipment to perform CLASI-FISH. Therefore, having a FISH on-chip device with such capabilities is probably beyond the horizon in the next few years, but would certainly be a breakthrough in this field.

Finally, and despite the great technological challenges described above, the limiting steps for getting these microfluidic products into the diagnostic market are probably the high cost of some chips, not competitive with the routine methods already implemented in laboratories, and the lack of validation. In fact, the complex regulations for validating clinical or food safety products, as well as the requirements of appropriate quality management systems for producing such devices, are important barriers for research centers that typically do not have resources allocated to such tasks. Development beyond the academic laboratories towards a marketable product, developed in concert with end-users, and engagement with diagnostic companies and regulators are required to push this field further.

## References

[1] Gall JG, Pardue ML (1969). Formation and detection of RNA-DNA hybrid molecules in cytological preparations. Proc Natl Acad Sci.

[2] Manning JE, Hershey ND, Broker TR, Pellegrini M, Mitchell HK, Davidson N (1975). A new method of in situ hybridization. Chromosoma.

[3] Bauman JGJ, Wiegant J, Borst P, van Duijn P (1980). A new method for fluorescence microscopical localization of specific DNA sequences by in situ hybridization of fluorochrome-labelled RNA. Exp Cell Res.

[4] Ratan ZA, Bin Zaman S, Mehta V, Haidere MF, Runa NJ, Akter N (2017). Application of fluorescence in situ hybridization (FISH) technique for the detection of genetic aberration in medical science. Cureus.

[5] Frickmann H, Zautner AE, Moter A, Kikhney J, Hagen RM, Stender H, Poppert S (2017). Fluorescence in situ hybridization (FISH) in the microbiological diagnostic routine laboratory: a review. Crit Rev Microbiol.

[6] ThermoFisher: fluorescence in situ hybridization (FISH). https://www.thermofisher.com/uk/en/home/life-science/cell-analysis/cellular-imaging/in-situ-hybridization-ish/fluorescence-in-situ-hybridization-fish.html. Accessed 10 June 2019

[7] Abnova: fluorescence in situ hybridization (FISH) probes. http://www.abnova.com/support/resources.asp?switchfunctionid=%7BB4285500-DB85-435D-BE02-2BF420D5C70D%7D. Accessed 12 June 2019

[8] Cytocell ltd: myProbes-custom probes. https://www.cytocell.com/custom-probes. Accessed 12 June 2019

[9] Biomode: PNA FISH. https://biomode-sa.com/technology/. Accessed 12 June 2019

[10] O’Connor C (2005). Fluorescence in situ hybridization. Nat Methods.

[11] Meloni AM, Peier AM, Haddad FS, Powell IJ, Block AW, Huben RP, Todd I, Potter W, Sandberg AA (1993). A new approach in the diagnosis and follow-up of bladder-cancer—FISH analysis of urine, bladder washings, and tumors. Cancer Genet Cytogenet.

[12] Hsu J-T, Chen T-C, Tseng J-H, Chiu C-T, Liu K-H, Yeh C-N, Hwang T-L, Jan Y-Y, Yeh T-S (2011). Impact of HER-2 overexpression/amplification on the prognosis of gastric cancer patients undergoing resection: a single-center study of 1036 patients. Oncologist.

[13] Dendukuri N, Khetani K, McIsaac M, Brophy J (2007). Testing for HER2-positive breast cancer: a systematic review and cost-effectiveness analysis. Can Med Assoc J.

[14] Larson DR, Singer RH, Zenklusen D (2009). A single molecule view of gene expression. Trends Cell Biol.

[15] DeLong EF, Wickham GS, Pace NR (1989). Phylogenetic stains: ribosomal RNA-based probes for the identification of single cells. Science.

[16] Amann RI, Ludwig W, Schleifer KH (1995). Phylogenetic identification and in situ detection of individual microbial cells without cultivation. Microbiol Rev.

[17] Amann R, Fuchs BM (2008). Single-cell identification in microbial communities by improved fluorescence in situ hybridization techniques. Nat Rev Microbiol.

[18] Vogel CL, Cobleigh MA, Tripathy D, Gutheil JC, Harris LN, Fehrenbacher L, Slamon DJ, Murphy M, Novotny WF, Burchmore M, Shak S, Stewart SJ, Press M (2002). Efficacy and safety of trastuzumab as a single agent in first-line treatment of HER2-overexpressing metastatic breast cancer. J Clin Oncol.

[19] Carlson B (2008). HER2 tests: how do we choose?. Biotechnol Healthc.

[20] Wolff AC, Hammond MEH, Hicks DG, Dowsett M, McShane LM, Allison KH, Allred DC, Bartlett JMS, Bilous M, Fitzgibbons P, Hanna W, Jenkins RB, Mangu PB, Paik S, Perez EA, Press MF, Spears PA, Vance GH, Viale G, Hayes DF (2013). Recommendations for human epidermal growth factor receptor 2 testing in breast cancer: american society of clinical oncology/college of american pathologists clinical practice guideline update. J Clin Oncol.

[21] Moelans CB, de Weger RA, Van der Wall E, van Diest PJ (2011). Current technologies for HER2 testing in breast cancer. Crit Rev Oncol Hematol.

[22] Cerqueira L, Azevedo NF, Almeida C, Jardim T, Keevil CW, Vieira MJ (2008). DNA mimics for the rapid identification of microorganisms by fluorescence in situ hybridization (FISH). Int J Mol Sci.

[23] Prudent E, Raoult D (2019). Fluorescence in situ hybridization, a complementary molecular tool for the clinical diagnosis of infectious diseases by intracellular and fastidious bacteria. FEMS Microbiol Rev.

[24] Almeida C, Azevedo NF, Fernandes RM, Keevil CW, Vieira MJ (2010). Fluorescence in situ hybridization method using a peptide nucleic acid probe for identification of salmonella spp. in a broad spectrum of samples. Appl Environ Microbiol.

[25] Rohde A, Hammerl JA, Appel B, Dieckmann R, Al Dahouk S (2015). FISHing for bacteria in food—a promising tool for the reliable detection of pathogenic bacteria?. Food Microbiol.

[26] Kebschull JM, Zador AM (2015). Sources of PCR-induced distortions in high-throughput sequencing data sets. Nucleic Acids Res.

[27] Wilson IG (1997). Inhibition and facilitation of nucleic acid amplification. Appl Environ Microbiol.

[28] Acinas SG, Sarma-Rupavtarm R, Klepac-Ceraj V, Polz MF (2005). PCR-induced sequence artifacts and bias: insights from comparison of two 16S rRNA clone libraries constructed from the same sample. Appl Environ Microbiol.

[29] Arora A, Simone G, Salieb-Beugelaar GB, Kim JT, Manz A (2010). Latest developments in micro total analysis systems. Anal Chem.

[30] Kovarik ML, Gach PC, Ornoff DM, Wang YL, Balowski J, Farrag L, Allbritton NL (2012). Micro total analysis systems for cell biology and biochemical assays. Anal Chem.

[31] Culbertson CT, Mickleburgh TG, Stewart-James SA, Sellens KA, Pressnall M (2014). Micro total analysis systems: fundamental advances and biological applications. Anal Chem.

[32] Patabadige DEW, Jia S, Sibbitts J, Sadeghi J, Sellens K, Culbertson CT (2016). Micro total analysis systems: fundamental advances and applications. Anal Chem.

[33] Kwasny D, Vedarethinam I, Shah P, Dimaki M, Silahtaroglu A, Tumer Z, Svendsen WE (2012). Advanced microtechnologies for detection of chromosome abnormalities by fluorescent in situ hybridization. Biomed Microdevice.

[34] Sato K (2015). Microdevice in cellular pathology: microfluidic platforms for fluorescence in situ hybridization and analysis of circulating tumor cells. Anal Sci.

[35] Huber D, Voith von Voithenberg L, Kaigala GV (2018). Fluorescence in situ hybridization (FISH): history, limitations and what to expect from micro-scale FISH?. Micro Nano Eng.

[36] Hasnain S, Jacobson MP, Bandyopadhyay P (2014). A comparative Brownian dynamics investigation between small linear and circular DNA: scaling of diffusion coefficient with size and topology of DNA. Chem Phys Lett.

[37] Sieben VJ, Marun CSD, Pilarski PM, Kaigala GV, Pilarski LM, Backhouse CJ (2007). FISH and chips: chromosomal analysis on microfluidic platforms. IET Nanobiotechnol.

[38] Zanardi A, Bandiera D, Bertolini F, Corsini CA, Gregato G, Milani P, Barborini E, Carbone R (2010). Miniaturized FISH for screening of onco-hematological malignancies. Biotechniques.

[39] Zanardi A, Barborini E, Carbone R, Jenkins G, Mansfield CD (2013). microFIND^®^ approach to fluorescent in situ hybridization (FISH). Microfluidic diagnostics: methods and protocols.

[40] Ho SSY, Chua C, Gole L, Biswas A, Koay E, Choolani M (2012). Same-day prenatal diagnosis of common chromosomal aneuploidies using microfluidics-fluorescence in situ hybridization. Prenat Diagn.

[41] Mughal F, Baldock SJ, Karimiani EG, Telford N, Goddard NJ, Day PJR (2014). Microfluidic channel-assisted screening of hematopoietic malignancies. Genes Chromosom Cancer.

[42] Mottet G, Perez-Toralla K, Tulukcuoglu E, Bidard F-C, Pierga J-Y, Draskovic I, Londono-Vallejo A, Descroix S, Malaquin L, Louis Viovy J (2014). A three dimensional thermoplastic microfluidic chip for robust cell capture and high resolution imaging. Biomicrofluidics.

[43] Perez-Toralla K, Mottet G, Guneri ET, Champ J, Bidard F-C, Pierga J-Y, Klijanienko J, Draskovic I, Malaquin L, Viovy J-L, Descroix S (2015). FISH in chips: turning microfluidic fluorescence in situ hybridization into a quantitative and clinically reliable molecular diagnosis tool. Lab Chip.

[44] Dickson MN, Tsinberg P, Tang ZL, Bischoff FZ, Wilson T, Leonard EF (2011). Efficient capture of circulating tumor cells with a novel immunocytochemical microfluidic device. Biomicrofluidics.

[45] Mayer JA, Pham T, Wong KL, Scoggin J, Sales EV, Clarin T, Pircher TJ, Mikolajczyk SD, Cotter PD, Bischoff FZ (2011). FISH-based determination of HER2 status in circulating tumor cells isolated with the microfluidic CEE™ platform. Cancer Genet.

[46] Krishnamurthy S, Bischoff F, Mayer JA, Wong K, Pham T, Kuerer H, Lodhi A, Bhattacharyya A, Hall C, Lucci A (2013). Discordance in HER2 gene amplification in circulating and disseminated tumor cells in patients with operable breast cancer. Cancer Med.

[47] Vedarethinam I, Shah P, Dimaki M, Tumer Z, Tommerup N, Svendsen WE (2010). Metaphase FISH on a chip: miniaturized microfluidic device for fluorescence in situ hybridization. Sensors.

[48] Shah P, Vedarethinam I, Kwasny D, Andresen L, Skov S, Silahtaroglu A, Tumer Z, Dimaki M, Svendsen WE (2011). FISHprep: a novel integrated device for metaphase FISH sample preparation. Micromachines.

[49] Shah P, Vedarethinam I, Kwasny D, Andresen L, Dimaki M, Skov S, Svendsen WE (2011). Microfluidic bioreactors for culture of non-adherent cells. Sens Actuators B Chem.

[50] Liu Y, Kirkland B, Shirley J, Wang Z, Zhang P, Stembridge J, Wong W, Takebayashi S-i, Gilbert DM, Lenhert S, Guan J (2013). Development of a single-cell array for large-scale DNA fluorescence in situ hybridization. Lab Chip.

[51] Lee DS, Lee JH, Min HC, Kim TY, Oh BR, Kim HY, Lee JY, Lee CK, Chun HG, Kim HC (2007). Application of high throughput cell array technology to FISH: investigation of the role of deletion of p16 gene in leukemias. J Biotechnol.

[52] Matsunaga T, Hosokawa M, Arakaki A, Taguchi T, Mori T, Tanaka T, Takeyama H (2008). High-efficiency single-cell entrapment and fluorescence in situ hybridization analysis using a poly(dimethylsiloxane) microfluidic device integrated with a black poly(ethylene terephthalate) micromesh. Anal Chem.

[53] Kurz CM, Maurer A, Thees K, Schillberg S, Velten T, Thielecke H (2011). Impedance-controlled cell entrapment using microhole-array chips allows the isolation and identification of single, highly productive cells. Sens Actuators B Chem.

[54] Kurz CM, von der Moosdijk S, Thielecke H, Velten T, IEEE (2011) Towards a cellular multi-parameter analysis platform: fluorescence in situ hybridization (FISH) on microhole-array chips. In: 2011 annual international conference of the IEEE Engineering in Medicine and Biology Society, IEEE Engineering in Medicine and Biology Society conference proceedings, pp 8408–8411 10.1109/IEMBS.2011.6092074 22256298

[55] Riahi R, Gogoi P, Sepehri S, Zhou Y, Handique I, Godsey J, Wang YX (2014). A novel microchannel-based device to capture and analyze circulating tumor cells (CTCs) of breast cancer. Int J Oncol.

[56] Gogoi P, Sepehri S, Zhou Y, Gorin MA, Paolillo C, Capoluongo E, Gleason K, Payne A, Boniface B, Cristofanilli M, Morgan TM, Fortina P, Pienta KJ, Handique K, Wang Y (2016). Development of an automated and sensitive microfluidic device for capturing and characterizing circulating tumor cells (CTCs) from clinical blood samples. PLoS One.

[57] Shaffer SM, Joshi RP, Chambers BS, Sterken D, Biaesch AG, Gabrieli DJ, Li Y, Feemster KA, Hensley SE, Issadore D, Raj A (2015). Multiplexed detection of viral infections using rapid in situ RNA analysis on a chip. Lab Chip.

[58] Zhang Q, Zhu L, Feng HH, Ang S, Chau FS, Liu WT (2006). Microbial detection in microfluidic devices through dual staining of quantum dots-labeled immunoassay and RNA hybridization. Anal Chim Acta.

[59] Ferreira AM, Cruz-Moreira D, Cerqueira L, Miranda JM, Azevedo NF (2017). Yeasts identification in microfluidic devices using peptide nucleic acid fluorescence in situ hybridization (PNA-FISH). Biomed Microdevices.

[60] Vincent ME, Liu WS, Haney EB, Ismagilov RF (2010). Microfluidic stochastic confinement enhances analysis of rare cells by isolating cells and creating high density environments for control of diffusible signals. Chem Soc Rev.

[61] Liu WS, Kim HJ, Lucchetta EM, Du WB, Ismagilov RF (2009). Isolation, incubation, and parallel functional testing and identification by FISH of rare microbial single-copy cells from multi-species mixtures using the combination of chemistrode and stochastic confinement. Lab Chip.

[62] Packard MM, Shusteff M, Alocilja EC (2012). Microfluidic-based amplification-free bacterial DNA detection by dielectrophoretic concentration and fluorescent resonance energy transfer assisted in situ hybridization (FRET-ISH). Biosensors.

[63] Sieben VJ, Debes-Marun CS, Pilarski LM, Backhouse CJ (2008). An integrated microfluidic chip for chromosome enumeration using fluorescence in situ hybridization. Lab Chip.

[64] Skelley AM, Scherer JR, Aubrey AD, Grover WH, Ivester RHC, Ehrenfreund P, Grunthaner FJ, Bada JL, Mathies RA (2005). Development and evaluation of a microdevice for amino acid biomarker detection and analysis on Mars. Proc Natl Acad Sci USA.

[65] Tai CH, Ho CL, Chen YL, Chen W, Lee GB (2013). A novel integrated microfluidic platform to perform fluorescence in situ hybridization for chromosomal analysis. Microfluid Nanofluid.

[66] Liu P, Meagher RJ, Light YK, Yilmaz S, Chakraborty R, Arkin AP, Hazen TC, Singh AK (2011). Microfluidic fluorescence in situ hybridization and flow cytometry (μFlowFISH). Lab Chip.

[67] Kao KJ, Tai CH, Chang WH, Yeh TS, Chen TC, Lee GB (2015). A fluorescence in situ hybridization (FISH) microfluidic platform for detection of HER2 amplification in cancer cells. Biosens Bioelectron.

[68] Søe MJ, Okkels F, Sabourin D, Alberti M, Holmstrøm K, Dufva M (2011). HistoFlex—a microfluidic device providing uniform flow conditions enabling highly sensitive, reproducible and quantitative in situ hybridizations. Lab Chip.

[69] Nguyen HT, Trouillon R, Matsuoka S, Fiche M, de Leval L, Bisig B, Gijs MAM (2017). Microfluidics-assisted fluorescence in situ hybridization for advantageous human epidermal growth factor receptor 2 assessment in breast cancer. Lab Invest.

[70] Nguyen HT, Bernier LS, Jean AM, Trouillon R, Gijs MAM (2017). Microfluidic-assisted chromogenic in situ hybridization (MA-CISH) for fast and accurate breast cancer diagnosis. Microelectron Eng.

[71] Nguyen HT, Dupont LN, Cuttaz EA, Jean AM, Trouillon R, Gijs MAM (2018). Breast cancer HER2 analysis by extra-short incubation microfluidics-assisted fluorescence in situ hybridization (ESIMA FISH). Microelectron Eng.

[72] Nguyen HT, Migliozzi D, Bisig B, de Leval L, Gijs MAM (2019). High-content, cell-by-cell assessment of HER2 overexpression and amplification: a tool for intratumoral heterogeneity detection in breast cancer. Lab Invest.

[73] Kaigala GV, Lovchik RD, Drechsler U, Delamarche E (2011). A vertical microfluidic probe. Langmuir.

[74] Ostromohov N, Bercovici M, Kaigala GV (2016). Delivery of minimally dispersed liquid interfaces for sequential surface chemistry. Lab Chip.

[75] Huber D, Autebert J, Kaigala GV (2016). Micro fluorescence in situ hybridization (µFISH) for spatially multiplexed analysis of a cell monolayer. Biomed Microdevices.

[76] Huber D, Kaigala GV (2018). Rapid micro fluorescence in situ hybridization in tissue sections. Biomicrofluidics.

[77] Ostromohov N, Huber D, Bercovici M, Kaigala GV (2018). Real-time monitoring of fluorescence in situ hybridization kinetics. Anal Chem.

[78] Kubota K (2013). CARD-FISH for environmental microorganisms: technical advancement and future applications. Microbes Environ.

[79] Pratscher J, Stichternoth C, Fichtl K, Schleifer KH, Braker G (2009). Application of recognition of individual genes-fluorescence in situ hybridization (RING-FISH) to detect nitrite reductase genes (nirK) of denitrifiers in pure cultures and environmental samples. Appl Environ Microbiol.

[80] Valm AM, Welch JLM, Rieken CW, Hasegawa Y, Sogin ML, Oldenbourg R, Dewhirst FE, Borisy GG (2011). Systems-level analysis of microbial community organization through combinatorial labelling and spectral imaging. Proc Natl Acad Sci.

[81] Biocept https://biocept.com. Accessed 13 June 2019

